# Quantification of Neocortical Slice Diffusion Characteristics Using Pharmacokinetic and Pharmacodynamic Modelling

**DOI:** 10.1155/2013/759640

**Published:** 2013-09-05

**Authors:** Logan J. Voss, Claudia van Kan, James W. Sleigh

**Affiliations:** ^1^Anaesthesia Department, Waikato District Health Board, Pembroke St, Hamilton 3240, New Zealand; ^2^University of Amsterdam, 1012 ZA Amsterdam, The Netherlands; ^3^Department of Anaesthesiology, University of Auckland, Auckland 1142, New Zealand

## Abstract

Pharmacological brain slice experiments are complicated by the need to ensure adequate drug delivery deep into the healthy layers of the tissue. Because tissue slices have no blood supply, this is achieved solely by passive drug diffusion. The aim of this study was to determine whether pharmacokinetic/pharmacodynamic (PKPD) modeling could be adapted to estimate drug diffusion times in neocortical brain slices. No-magnesium seizure-like event (SLE) activity was generated in 41 slices (400 **μ**m). Two anesthetic agents, etomidate (24 **μ**M, *n* = 14) and thiopental (250 **μ**M, *n* = 14), and magnesium ions (*n* = 13) were delivered to effect reversible reductions in SLE frequency. Concentration-effect hysteresis loops were collapsed using a first order rate constant model and equilibrium half-lives (t_1/2_K_e0_) derived. The t_1/2_K_e0_ values obtained were consistent with expectations. The median (range) t_1/2_K_e0_ of 83.1 (19.4–330.1) min for etomidate is in keeping with its known slow diffusion into brain slice tissue. Values for etomidate and thiopental (111.8 (27.8–198.0) min) were similar, while magnesium had a significantly faster equilibration rate (t_1/2_K_e0_ of 26.1 (8.6–77.0) min) compared to the anesthetics, as expected for a simple ion. In conclusion, PKPD modeling is a simple and practical method that can be applied to brain slice experiments for investigating drug diffusion characteristics.

## 1. Introduction

Characterisation of drug effects in *in vitro *brain slice experiments is complicated by the absence of a blood supply to the tissue. This means that drugs must be delivered to the tissue by passive diffusion via the perfusion solution. Because substances diffuse at different rates according to their physicochemical properties, it can be difficult to know how long to expose the tissue in order to achieve drug penetration into the healthiest layers of the slice—approximately the middle 100 *μ*m for a 400 *μ*m thick slice [1]. For drugs that diffuse slowly, there is a risk that a negative result will be spuriously attributed to an inactive drug, when in fact it never reached its site of action in the tissue.

Directly measuring drug concentrations in tissue slicesnecessitates methodologies that are time consuming and complex and cannot realistically be applied to each and every drug one may want to test. This motivated us to explore alternative, simpler approaches for estimating drug diffusion in brain slice tissue. To this end, we have borrowed a pharmacological technique known as pharmacokinetic/pharmacodynamic (PKPD) modelling, which is used clinically to describe the relationship between the time course of drug effect and the delivered dose. When anesthetic drugs are delivered to the circulation, for example, there is a measurable time delay before the subject becomes unconscious. The length of the delay is related to the time taken for the drug to diffuse from the circulation to the “effect site” in the brain. By applying Fick's law of diffusion (stating that the rate of equilibration of a substance is proportional to its concentration gradient), it is possible to calculate the equilibration half-time (*t*_1/2_ *K*_*e*0_)—a measure of the time required for a substance to reach half of its equilibrated concentration at the tissue recording site.

In this study we have applied these principles to quantify the rate at which anesthetic drugs diffuse into neocortical slice tissue. One of these (etomidate) was chosen because its diffusion characteristics in brain tissue have been carefully investigated [2], allowing a direct comparison with the results of this study. We also compared the anesthetic drugs to magnesium, a simple ion that we hypothesised should diffuse at a faster rate.

## 2. Materials and Methods

### 2.1. Ethics Statement

All experimental procedures were approved by the Waikato Ethics Committee at Waikato University, Hamilton, Waikato, New Zealand (ethics approval number 836).

### 2.2. Artificial Cerebrospinal Fluid (aCSF) Solutions

The solutions were made with double distilled water and stored at 1–4°C for no longer than 7 days. All solutions were saturated with carbogen (95% O_2_; 5% CO_2_) for at least 15 minutes prior to use. Three solutions were used.

1. “*Protective*”: aCSF for brain extraction and tissue slicing, containing 92.7 mM NaCl, 3 mM KCl, 19 mM MgCl_2_, 0 mM CaCl_2_, 1.2 mM NaH_2_PO_4_, 24 mM NaHCO_3_, and 25 mM D-glucose [3].
2. “*No-magnesium*”: aCSF lacking magnesium ions, containing 124 mM NaCl, 5 mM KCl, 2 mM CaCl_2_, 1.25 mM NaH_2_PO_4_, 26 mM NaHCO_3_, and 10 mM D-glucose.
3. “*Normal*”: aCSF containing magnesium ions, composed of 125 mM NaCl, 2.5 mM KCl, 1 mM MgCl_2_, 2 mM CaCl_2_, 1.25 mM NaH_2_PO_4_, 26 mM NaHCO_3_, and 10 mM D-glucose.

### 2.3. Tissue Preparation

Mouse-neocortical brain slices were obtained from adult wild-type (129SV) mice, both female and male. Prior to decapitation and brain dissection, the mice were anesthetised with carbon dioxide (CO_2_). The cerebrum was removed and placed into ice-cold protective aCSF, made according to Nowak and Bullier [3]. A vibratome (Campden Instruments, UK) was used to slice the brain in 400 *μ*M thick coronal sections between Bregma −1 mm and −5 mm. Each slice was placed into carbogenated no-magnesium aCSF solution to recover for at least an hour prior to recording at room temperature (approximately 28°C). For experimental recording, each slice was transferred to a recording bath (Tissue Recording System, Kerr Scientific Instruments, New Zealand) perfused with carbogenated no-magnesium aCSF solution at a gravity-fed flow rate of 6.0 mL/min.

### 2.4. Extracellular Field Potential Recording

Extracellular field potentials were recorded from a 50 *μ*m Teflon-coated tungsten electrode, referenced to a silver/silver-chloride electrode positioned in the recording bath. The recording electrode was positioned at a depth of approximately 200 *μ*m (i.e., the middle of the slice) in the cerebral cortex, with no particular cortical location targeted. The data was recorded with a 1000x gain, low- and high-pass filtered at 1000 Hz, and 1.0 Hz respectively (Model 1800 AC amplifier, A-M Systems, USA), and sampled at a frequency of 5000 samples/second (Power 1401, Cambridge Electronic Designs, UK). Mains filtering at 50 Hz was not required because the experiments were conducted within an electrically shielded room. The data was saved for analysis using Matlab (Version 7.3.0.267 (R2006b), The MathWorks Inc., Natick, MA, USA).

### 2.5. Experimental Procedures

Spontaneous seizure-like event (SLE) activity was recorded for at least 10 minutes prior to anesthetic delivery. Thereafter the anesthetic of choice was delivered to the tissue using the same gravity-fed perfusion system, resulting in a gradual increase in concentration within the recording bath. Anesthetic delivery was continued for 15 minutes, followed by washout with drug-free no-magnesium aCSF. No-magnesium aCSF perfusion was continued until the frequency of SLEs returned to baseline. 

Two anesthetics were tested: etomidate (24 *μ*M, *n* = 14 from 4 animals) and thiopental (250 *μ*M, *n* = 14 from 4 animals). Drug dosage was based on previous cortical slice experiments using etomidate [4] and the relative clinical potencies of the two agents [5]. The anesthetic solutions were made by adding the appropriate amount of each drug directly to precarbogenated aCSF immediately prior to perfusion. 

For comparison with the anesthetic drugs, we also tested the diffusion of magnesium ions (*n* = 13 from 2 animals). Slices were perfused with magnesium-containing normal aCSF (which eliminates SLE activity) for 15 minutes, followed by washout of magnesium with no-magnesium aCSF until SLE activity returned to baseline levels. The data was analysed in an identical fashion to the anesthetic agents (see below).

The SLE time courses were used to plot the relationship between drug effect (SLE frequency) and drug concentration. The changes in anesthetic and magnesium ion concentrations with time were not measured directly in this study. Rather, an estimate of the concentration time course was obtained in a preliminary experiment by measuring the real-time changes in bath solution conductivity during perfusion of an 0.1 mM solution of sodium chloride. The salt solution was perfused into the bath from a starting concentration of 0 mM, using the same flow parameters as for the experimental protocols. Because solution conductivity increases approximately linearly with the increase in concentration of salt in solution [6], the time course of conductivity changes give an estimate of the time-course of concentration change. We made the assumption that the time course for perfusion of the salt solution into the bath would be similar to that for the anesthetics.

### 2.6. Analysis and Statistical Tests

**Figure 1:**
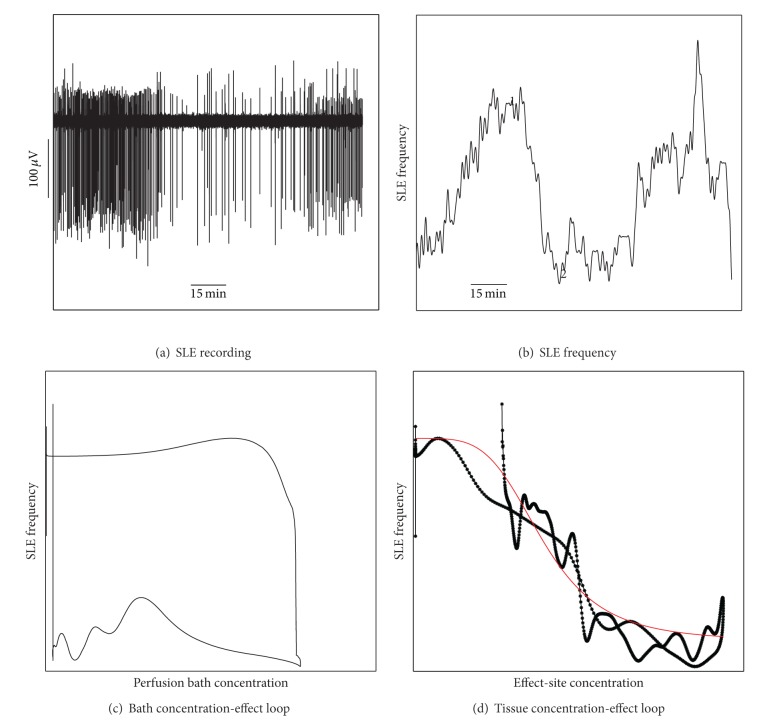
Showing the sequence of analysis from (a) raw recording of SLE activity, (b) time-frequency plot of SLE activity, (c) SLE frequency versus bath anesthetic concentration relationship, and (d) collapsed hysteresis loop with fitted inhibitory *E*_max⁡_ curve, representing the tissue effect-site concentration relationship.

The sequence of analysis steps is shown in Figure 1. Concentration-effect plots displayed the anticipated hysteresis loops (Figure 1(c)), reflecting (1) the time delay between delivery of the drug in the perfusion solution and its diffusion to the site of action in the tissue and (2) the reverse delay between removal of the drug from the perfusion solution and its washout from the tissue. We used a standard pharmacokinetic/pharmacodynamic (PKPD) model to “collapse” this loop (Figure 1(d)) in order to obtain the first order rate constant *K*_*e*0_, according to

(1) $$\begin{array}{c} \frac{{dC}_{\text{eff}}}{dt}=K_{e0}\left(C_{p}-C_{\text{eff}}\right), \end{array}$$

where *C*_*p*_ is the aCSF concentration of the drug, *C*_eff_ is the concentration at the effect site in the tissue, and *K*_*e*0_ is the first order rate constant for movement of drug into the tissue. The *C*_eff_ was estimated by iteratively running the aforementioned model with a series of *K*_*e*0_ steps. For each iteration, a nonlinear inhibitory sigmoid *E*_max⁡_ curve was fitted to the data (Figure 1(d)) by the following equation:

(2) $$\begin{array}{c} \text{Effect}=E_{\max}-\left(E_{\max}-E_{\min}\right)\times \frac{C_{\text{eff}}^{\gamma }}{{EC}_{50}^{\gamma }+C_{\text{eff}}^{\gamma }}, \end{array}$$

where Effect is the SLE frequency, the *E*_max⁡_ and *E*_min⁡_ are the maximum and minimum Effects for each slice, *EC*_50_ is the anesthetic concentration at which Effect is midway between this maximum and minimum, and gamma (*γ*) describes the slope of the concentration-response relationship. *K*_*e*0_ was determined from the iteration yielding the greatest coefficient of determination (*R* ^2^) for measured and modelled Effect for each slice. *K*_*e*0_ was converted into *K*_*e*0_ half-life (*t*_1/2_ *K*_*e*0_), according to the relationship

(3) $$\begin{array}{c} t_{1/2}K_{e0}=\frac{\ln2}{K_{e0}}. \end{array}$$

All data are presented as median (range). Data normality was assessed using the Kolmogorov-Smirnov test and statistical tests applied accordingly. A *P* value <0.05 was considered significant.

## 3. Results

### 3.1. Seizure-Like Event (SLE) Activity

No-magnesium SLE activity was generated in 41 slices from 14 mice. SLEs were characterized by an initial large population depolarization, followed occasionally by a 4–7 Hz oscillation of approximately three seconds. The baseline median (range) SLE frequency for each test condition was 2.5 (1.4–3.2), 3.5 (1.1–6.5), and 2.4 (1.0–3.9) events/min for etomidate, thiopental, and magnesium, respectively. In each case, delivery of the test compound reliably effected a reduction in SLE frequency to 1.6 (0.4–3.2), 0.9 (0.4–2.6), and 0.5 (0.2–1.6) events/min, respectively, before returning to baseline levels after drug washout. There was no statistically significant difference in baseline SLE frequency across the three test conditions (*P* = 0.13, Kruskal-Wallis test), and SLE frequency following drug washout was similar to baseline in all cases (*P* = 0.82, Kruskal-Wallis test). An example of the pattern of SLE activity from one slice is shown in Figure 1(a). 

### 3.2. PKPD Parameters

*t*_1/2_ *K*_*e*0_ values calculated for each anesthetic and for the magnesium aCSF experiments are shown in Table 1. A value of 83.1 (19.4–330.1) min for etomidate represents its equilibration half-time. In other words, after 83 minutes, etomidate had reached half of its equilibrated concentration at the site of recording (in the middle of the slice). *t*_1/2_ *K*_*e*0_ values were similar for the two anesthetics but significantly shorter for magnesium (26.1 (8.6–77.0) min, *P* < 0.01 for comparison with both thiopental and etomidate, Kruskal-Wallis test). This was consistent with the expectation that magnesium ions would diffuse rapidly into the slice tissue. 

Additional parameters, *EC*_50_ and gamma, were derived from the inhibitory *E*_max⁡_ curves, relating to the potency of the drug and speed of onset/offset, respectively (Table 1). The only difference of note was a higher *EC*_50_ value for etomidate compared to the other agents, suggesting that etomidate effected a lesser reduction in event frequency. This is confirmed by the SLE frequency data showing that event frequency at maximum etomidate effect was higher compared to both magnesium and thiopental (*P* < 0.001 and *P* < 0.05, resp., one-way ANOVA). 

## 4. Discussion

The aim of this study was to determine whether an analytical technique carried over from clinical pharmacological studies could be adapted and applied to cortical slices for estimating drug diffusion times. We were motivated by the need for a simple and practical method of determining the minimum time required for a given drug to reach effective concentrations within the depth of slice tissue. To achieve this, we estimated the rate of equilibration between aCSF and the cortical slice effect site by calculating the *t*_1/2_ *K*_*e*0_ for two anesthetic drugs and magnesium ions. 

The results are encouraging on the basis of three comparisons. Firstly, the long equilibration time calculated in this study for etomidate (*t*_1/2_ *K*_*e*0_ of 83 min) is consistent with analytical studies of the same drug showing that it takes upwards of 100 minutes for etomidate to reach equilibrium in the middle of a 400 *μ*m thick brain slice [2]. Secondly, values for etomidate and thiopental were not significantly different, which is in agreement with *in vivo* PKPD studies showing that the equilibration delays for these agents are similar [7–11]. Thirdly, the much shorter *t*_1/2_ *K*_*e*0_ for magnesium (approximately 30 minutes) matches the expectation that a simple ion should diffuse much faster than the more chemically complex anesthetic drugs. 

The diffusion time was substantially longer in the slice model compared to *in vivo*, with *t*_1/2_ *K*_*e*0_ values for etomidate (*t*_1/2_ *K*_*e*0_ 1.5–2.7 min) and thiopental (*t*_1/2_ *K*_*e*0_ 1.2 min) more than an order of magnitude shorter than calculated for the same agents in this study [7–11]. This was anticipated because the diffusion distance in the slice is 5–10 times greater than *in vivo *[12]. The intercapillary distance in the normal mammalian cerebral cortex is in the order of 50–60 *μ*m [12], meaning that the maximum diffusion distance is 25–30 *μ*m. This compares to a diffusion distance of 100–200 *μ*m in the 400 *μ*m isolated cortical slice (depending on recording electrode depth). 

There was considerable variation in *t*_1/2_ *K*_*e*0_ values recorded from slice to slice. A proportion of this variability may be attributed to inconsistencies in inhibitory *E*_max⁡_ curve fitting due to discrepancies in the shape of the two halves of concentration-effect loops. This can result in slight mismatches between the two arms of the loop when collapsed, resulting in variability in curve fitting from test to test. One cause of such a mismatch could be a discrepancy in the frequency of SLE activity at the start of the recording compared to at the end. Another possible source of *t*_1/2_ *K*_*e*0_ variability could be small inconsistencies in electrode depth placement from slice to slice. While every effort was made to position the electrodes in the mid-layer of the slice, it was not possible to precisely measure electrode depth. As shown by Benkwitz et al., the rate of diffusion is faster near the top surface than deeper within the slice [2]. Thus, the concentration-time course will be somewhat affected by the depth position of the electrode. On the basis of this variability, a minimum of 10 repeat experiments is indicated in order to obtain a meaningful estimate of equilibration time. It is also important to note that the methods described account only for drug diffusion, not any additional delays to drug effect that could be associated with drug-receptor interactions and/or effect transduction pathways.

The anesthetic doses used in the present study were estimated to be approximately equipotent. Etomidate is 10 times more potent than thiopental [5]. On this basis, equipotent dosing using 24 *μ*M etomidate as a “standard” deep anesthetic dose [4] was estimated to be approximately 250 *μ*M for thiopental. Both anesthetics generated robust, repeatable, and reversible reductions in SLE frequency, confirming that the dosing was appropriate.

## 5. Conclusions

In conclusion, PKPD modelling confirms that the anesthetic drugs etomidate and thiopental diffuse slowly into cortical slice tissue and that this method is a useful tool for investigating drug diffusion characteristics in brain slice pharmacological studies.

## Figures and Tables

**Table 1:** PKPD parameter values: *t*_1/2_ *K*_*e*0_ (min), *EC*_50_, and gamma. All data are shown as median (range). The *EC*_50_ data is expressed as the proportion of the maximum concentration delivered.

	*t*_1/2_ *K*_*e*0_	*EC*_50_	Gamma
Normal aCSF (*n* = 13)	26.1 (8.6–77.0)	0.113^††^ (0.022–0.385)	3.780 (0.81–9.78)
Etomidate (*n* = 14)	83.1* (19.4–330.1)	0.334 (0.148–0.568)	3.031 (1.34–13.90)
Thiopental (*n* = 14)	111.8* (27.8–198.0)	0.247^†^ (0.017–0.442)	8.912 (0.89–31.57)

*P < 0.05, compared to normal aCSF, one-way ANOVA, Tukey-Kramer multiple comparisons test.

^††^ *P* < 0.05, compared to etomidate, one-way ANOVA, Tukey-Kramer multiple comparisons test.

^†^ *P* < 0.001, compared to etomidate, one-way ANOVA, Tukey-Kramer multiple comparisons test.

## References

[1] Cayce JM, Kao CC, Malphrus JD, Konrad PE, Mahadevan-Jansen A, Jansen ED (2010). Infrared neural stimulation of thalamocortical brain slices. *IEEE Journal on Selected Topics in Quantum Electronics*.

[2] Benkwitz C, Liao M, Laster MJ, Sonner JM, Eger EI, Pearce RA (2007). Determination of the EC50 amnesic concentration of etomidate and its diffusion profile in brain tissue. *Anesthesiology*.

[3] Nowak LG, Bullier J (1996). Spread of stimulating current in the cortical grey matter of rat visual cortex studied on a new in vitro slice preparation. *Journal of Neuroscience Methods*.

[4] Voss LJ, Brock M, Carlsson C, Steyn-Ross A, Steyn-Ross M, Sleigh JW (2012). Investigating paradoxical hysteresis effects in the mouse neocortical slice model. *European Journal of Pharmacology*.

[5] Kissin I, Motomura S, Aultman DF, Reves JG (1983). Inotropic and anesthetic potencies of etomidate and thiopental in dogs. *Anesthesia and Analgesia*.

[6] Zimmt WS, Odegaard N (1993). Conductivity measurements: a discussion and comparison of two instruments used to follow the removal of soluble salts from ceramics. *Western Association for Art Conservation*.

[7] de Paepe P, Belpaire FM, van Hoey G, Boon PA, Buylaert WA (1999). Influence of hypovolemia on the pharmacokinetics and the electroencephalographic effect of etomidate in the rat. *Journal of Pharmacology and Experimental Therapeutics*.

[8] Kaneda K, Yamashita S, Woo S, Han TH (2011). Population pharmacokinetics and pharmacodynamics of brief etomidate infusion in healthy volunteers. *Journal of Clinical Pharmacology*.

[9] Maitre PO, Buhrer M, Shafer SL, Stanski DR (1990). Estimating the rate of thiopental blood-brain equilibration using pseudo steady state serum concentrations. *Journal of Pharmacokinetics and Biopharmaceutics*.

[10] Stanski DR, Hudson RJ, Homer TD, Saidman LJ, Meathe E (1984). Pharmacodynamic modeling of thiopental anesthesia. *Journal of Pharmacokinetics and Biopharmaceutics*.

[11] Upton RN, Ludbrook GL (1999). A model of the kinetics and dynamics of induction of anaesthesia in sheep: variable estimation for thiopental and comparison with propofol. *British Journal of Anaesthesia*.

[12] Meier-Ruge W, Hunziker O, Schulz U, Tobler H-J, Schweizer A (1980). Stereological changes in the capillary network and nerve cells of the aging human brain. *Mechanisms of Ageing and Development*.

